# Lactate Dehydrogenase, Interleukin‐6, and 24 h Intra‐Abdominal Pressure Exhibit Early Predictive Value in Hypertriglyceridemic Pancreatitis Complicated by Acute Respiratory Distress Syndrome

**DOI:** 10.1155/emmi/8368299

**Published:** 2026-09-02

**Authors:** Kaiqiang Zhu, Bin Lu, Ranran Zhou, Lei Xu

**Affiliations:** ^1^ Department of Emergency, The Affiliated Hospital of Xuzhou Medical University, Xuzhou 221000, China, xzmc.edu.cn

**Keywords:** 24 h intra-abdominal pressure (IAP), acute respiratory distress syndrome (ARDS), hyperlipoidemia pancreatitis (HTG-AP), interleukin-6 (IL-6), lactate dehydrogenase (LDH), predictors

## Abstract

**Background:**

Hypertriglyceridemia‐induced acute pancreatitis (HTG‐AP) is increasingly recognized as a major cause of acute pancreatitis in China. Acute respiratory distress syndrome (ARDS), a severe and early complication, significantly worsens prognosis and increases intensive care unit (ICU) burden. This study aimed to identify early predictive markers of ARDS in patients with HTG‐AP, enabling timely risk stratification and clinical intervention to improve outcomes.

**Methods:**

A retrospective analysis was conducted on 111 patients who were diagnosed with HTG‐AP and admitted to the emergency ICU (EICU) between August 2022 and December 2024. Patients were categorized into ARDS (*n* = 61) and non‐ARDS (*n* = 50) groups according to the complications associated with ARDS throughout the progression of the illness. Clinical variables were compared between groups. The Boruta technique was used to choose predictors, and then multivariate logistic regression was used. To assess prediction ability, receiver operating characteristic (ROC) curve analysis was carried out. We performed internal validation using five‐fold cross‐validation and bootstrap resampling (1000 iterations) to evaluate the model’s capacity for generalization and stability. The clinical utility of the model was assessed using decision curve analysis (DCA).

**Results:**

Patients in the ARDS group had lower serum calcium (Ca^2+^) levels (*p* < 0.05) and longer ICU stays, as well as greater 24 h intra‐abdominal pressure (IAP), interleukin‐6 (IL‐6), lactate dehydrogenase (LDH), and lactate (Lac) levels (*p* < 0.05) compared to the non‐ARDS group. NEU, Lac, Cr, 24 h IAP, IL‐6, and LDH are important possible risk factors, according to the Boruta algorithm. Multivariate logistic regression analysis identified 24 h IAP, IL‐6, and LDH as independent predictors of concurrent ARDS (*p* < 0.05). The areas under the ROC curves (AUC) for 24 h IAP, IL‐6, and LDH were 0.796 (95% CI: 0.715–0.877), 0.770 (95% CI: 0.683–0.858), and 0.868 (95% CI: 0.880–0.936), respectively (*p* < 0.05). When combined, the predictive model yielded an AUC of 0.907 (95% CI: 0.849–0.957), with a specificity of 90.00% and a sensitivity of 78.70%, and demonstrated noninferior predictive performance to the existing scoring systems. With an average AUROC score of 0.890 from five‐fold cross‐validation and a bootstrap‐corrected *C*‐index of 0.894, the model shows good discriminatory power. The model’s clinical net benefit was verified by the DCA. Additionally, we have created a free online risk calculator for the general public (https://ardsprediction.streamlit.app/).

**Conclusions:**

LDH, IL‐6, and 24 h IAP are valuable early indicators of ARDS development in patients with HTG‐AP. The integrated predictive model demonstrates excellent diagnostic performance and can support early clinical decision‐making.

## 1. Introduction

Acute pancreatitis (AP) is a prevalent gastrointestinal condition that can affect various organ systems and progress rapidly, often leading to prolonged hospitalization and elevated mortality rates when it advances to severe AP (SAP) [1]. In recent years, hypertriglyceridemia‐induced AP (HTG‐AP) has surpassed alcohol as the second leading cause of AP in China, driven by rising living standards and changes in dietary habits [2].

Acute respiratory distress syndrome (ARDS) is the most commonly observed form of organ failure in AP patients [3]. ARDS has been reported in 4%–15% of AP patients [4], with its incidence rising to nearly one‐third in severe AP [5]. ARDS typically occurs early in the disease course, appearing within 2–7 days after the onset of pancreatic inflammation, and is linked to a significant mortality rate. Patients with ARDS often require a prolonged stay in the intensive care unit (ICU), which substantially increases economic and clinical burden [3]. A previous study revealed that prompt intervention during the early phases of ARDS can enhance clinical outcomes [6]. However, treatment strategies remain limited and primarily focus on supportive respiratory care, including oxygen therapy, mechanical ventilation, lung‐protective ventilation, prone positioning, and pulmonary recruitment maneuvers.

As a result, it is critical to quickly identify AP individuals who are susceptible to ARDS. The onset and progression of ARDS are significantly influenced by elevated levels of interleukin‐6 (IL‐6) and lactate dehydrogenase (LDH), which have been linked in numerous prior studies to increased disease severity, a higher incidence of complications, and an increased risk of death in patients with ARDS [7–9]. Additionally, we have shown in our clinical practice that individuals with early‐onset intra‐abdominal hypertension (IAH) have a greater frequency of ARDS and a tendency to have more severe disease. We hypothesize that LDH, IL‐6, and 24 h IAP are independent early predictors of ARDS in patients with HTG‐AP, and that a combined model integrating these three indicators can achieve highly accurate early risk assessment based on the aforementioned evidence and clinical observations.

While a number of prediction models have been developed to assess the severity of AP, such as the Sequential Organ Failure Assessment (SOFA), Acute Physiology and Chronic Health Evaluation (APACHE) II, and Bedside Index for Severity in Acute Pancreatitis (BISAP) scores, their application to HTG‐AP is still understudied, and none of them were specifically created to predict ARDS. Furthermore, the pathophysiological causes specific to HTG‐AP, such as IAH, cytokine storm, and lipid peroxidation‐induced tissue necrosis, are not captured by existing scoring systems, which mostly rely on general physiological measures. The creation of a composite predictive model that incorporates a mechanical signal (24 h IAP), an inflammatory marker (IL‐6), and a necrosis‐related marker (LDH) is what makes our study novel. With the potential to increase survival, shorten hospital stays, and maximize the use of healthcare resources, this multidomain approach offers an early, ARDS‐specific risk classification tool suited to the unique clinical course of HTG‐AP.

## 2. Materials and Methods

### 2.1. Patient Inclusion Criteria

The TRIPOD declaration for prediction model construction and the STROBE reporting requirements for observational studies were followed in the reporting of this study. Completed checklists are supplied in Supporting Information 1 (TRIPOD) and Supporting Information 2 (STROBE). A total of 111 HTG‐AP patients admitted to the emergency ICU (EICU) from August 2022 to December 2024 were retrospectively collected. This department uses a single electronic medical record system and standardized, consistent laboratory testing procedures. To minimize the interinstitutional differences in biomarker testing and case definitions that are common in multicenter studies, these standardized in‐house tests serve as the primary basis for selecting a single‐center study design. Rather than using convenience sampling, we included all eligible inpatients over the study period using a continuous, nonselective enrollment approach to minimize selection bias. The Affiliated Hospital of Xuzhou Medical University’s Ethics Committee authorized the study protocol (No. XYFY2025‐KL349‐01). The committee waived the requirement for informed consent in accordance with institutional standards because this was a retrospective analysis of pre‐existing clinical data with no more than minimal risk, the data were gathered from routine care, patient privacy was protected by anonymization, and obtaining consent would have been impossible due to discharge or loss to follow‐up. Patients presenting with symptoms of AP were admitted to the emergency department at a median of 24.00 (12.00, 48.00) h after symptom onset. Following the emergency evaluation lasting 4.68 (2.79, 8.14) h, 111 patients meeting the diagnostic criteria for HTG‐AP (TG ≥ 11.3 mmol/L) were admitted to the EICU. The development of ARDS was monitored daily, with a mean onset time of 59.07 ± 31.83 h from EICU admission to diagnosis. Baseline chest radiography and arterial blood gas analysis conducted at EICU admission excluded preexisting ARDS in all patients. Based on the presence or absence of ARDS during hospitalization, patients were stratified into 61 cases in the ARDS group and 50 cases. The diagnosis of AP followed the revised Atlanta Classification System (2012), while HTG‐AP was defined as AP with serum triglyceride (TG) ≥ 11.3 or 5.65–11.3 mmol/L with lipid‐rich celiac fluid. Moreover, the ARDS was diagnosed according to the 2024 Global Definition of Acute Respiratory Distress Syndrome [10].

### 2.2. Patient Exclusion Criteria

The exclusion criteria were as follows: preexisting respiratory failure before HTG‐AP onset, hospital stay of less than 24 h, having psychiatric disorders, concomitant autoimmune diseases or malignant neoplasms, and pregnancy. The planned 24 h IAP monitoring and 48 h laboratory test results for prognostic indicators were not available for patients who were discharged early or died, and some tests had not yet been completed and were canceled as a result of the patients’ early discharge, so patients who spent less than 24 h in the EICU after admission were not included in the primary analysis.

### 2.3. Data Collection and Processing

#### 2.3.1. Baseline Information

The baseline data obtained included demographic and clinical variables, including sex, age, chronic disease history (hypertension and diabetes mellitus), body mass index (BMI), frequency of previous pancreatitis episodes, smoking and alcohol consumption history, presence of comorbid diabetic ketoacidosis (DKA), administration of blood purification therapy, heart rate, and random blood glucose levels at admission. Furthermore, IAP was assessed utilizing the lateral bladder pressure technique and documented at 24 h following admission. Additional parameters included the ICU duration of stay and 24 h fluid balance. To ensure independence of observations, a 48 h IAP was excluded from the multivariate model due to temporal autocorrelation in sequential measurements within 48 h, with only a 24 h IAP retained as a predictor.

#### 2.3.2. Laboratory Test Indicators

The white blood cell count (WBC), neutrophil count (NEU), *C*‐reactive protein (CRP), erythrocyte pressure volume (HCT), procalcitonin (PCT), IL‐6, tumor necrosis factor‐*ɑ* (TNF‐*ɑ*), interferon‐*y* (IFN‐*y*), creatinine (Cr), total bilirubin (TBIL), LDH, TG, serum calcium (Ca^2+^), prothrombin time (PT), activated partial thromboplastin time (APTT), D‐dimer, and lactate (Lac) were collected from the patients in both groups within 24 h of admission. CRP and TG levels were collected at 48 h after admission. All patients underwent blood collection within 2 h of admission. Additionally, we detected cytokines using xMAP technology (Luminex, USA), and Nanjing AtomLife Technology Co., Ltd. supplied the necessary kits. We took 1‐2 mL of peripheral venous blood using EDTA‐anticoagulated tubes, processed the samples within an hour, and separated the plasma by centrifuging at 2000 rpm for 20 min at 4°C because some patients arrived at night and cytokine testing could not be done right away. After that, the samples were kept at 4°C or lower for testing the next day. The literature indicates that using this strategy can maintain steady levels of IL‐6, TNF‐*α*, and FN‐*γ* for up to 30 days [11].

#### 2.3.3. Scoring Systems

Within 24 h of admission, three recognized clinical scoring systems were computed for each patient: the BISAP, APACHE II, and SOFA. These scores were only utilized as a baseline for assessing the effectiveness of our new predictive model and for descriptive comparisons. For the following reasons, they were excluded from consideration as potential factors in the multivariate logistic regression model: (1) The variables included in APACHE II and BISAP either overlapped with our chosen predictors or lacked independent statistical significance in univariate analysis; (2) the respiratory component of the SOFA score partially overlaps with the diagnostic criteria for ARDS, which could result in circular reasoning; and (3) considering the sample size of this study, including all three scores in the regression analysis would increase the risk of overfitting. Due to duplication (such as overlap with SOFA) or insufficient validation in AP with hypertriglyceridemia, other scoring systems (qSOFA, MCTSI, and Ranson) were excluded from this investigation.

#### 2.3.4. Review of Data Integrity

For patients with HTG‐AP, our hospital’s Department of Emergency Medicine follows a standardized clinical protocol that includes routine testing for every parameter mentioned in this study (blood sampling within two hours of admission, 24 h IAP monitoring, 48 h follow‐up, etc.). After applying the inclusion and exclusion criteria, the final study cohort consisted of 111 patients. A randomly selected 20% sample (*n* = 22) was manually reviewed by two independent investigators, who cross‐referenced electronic data with original laboratory reports and medical records; no missing values were found. The data completeness of every variable was also verified through a systematic examination of the entire dataset. Due to incomplete data, three individuals whose hospital stays were shorter than twenty‐four hours were not included in the primary analysis.

### 2.4. Statistical Analysis

*R* (Version 4.5.1) and SPSS Version 25.0 were used for data analysis. Continuous variables with a normal distribution are presented as the mean ± standard deviation (SD) and were compared between groups using the independent samples *t*‐test, whereas nonnormally distributed variables are expressed as the median (interquartile range) and compared via the Mann‒Whitney *U* test. The normality of continuous variables was assessed using the Shapiro‒Wilk test. Nonnormally distributed variables (e.g., IL‐6 and LDH) were retained in their original scale without transformation, as logistic regression demonstrated robustness to moderate skewness. Categorical variables are presented as counts (percentages) (*n* (%)) and were evaluated using the chi‐square test. We ranked variables according to their significance for predicting ARDS using the Boruta method in order to reduce selection bias in the predictor selection process. The multiple logistic regression analysis then included variables that the Boruta algorithm deemed “important.” Binary logistic regression with backward elimination (probability of removal = 0.10) was used to identify factors associated with ARDS in HTG‐AP patients. Time‐dependence and multicollinearity (variance inflation factor [VIF] < 5) were assessed for the predictor variables in the final model. Predictive accuracy was evaluated via receiver operating characteristic (ROC) curve analysis, with optimal cutoff values determined by maximizing the Youden index (sensitivity + specificity − 1) to balance diagnostic accuracy. The area under the curve (AUC) was used to quantify performance, with higher values indicating superior prediction. Regression equations and an interactive online risk calculator were created based on the final model. To evaluate the model’s stability and capacity for generalization, 1000 bootstrap resamples and 5‐fold cross‐validation are used for internal validation. The Hosmer–Lemeshow test was used to determine goodness‐of‐fit. Calibration plots and estimations of the calibration slope and intercept are used to assess the calibration of the model. Decision curve analysis (DCA) was used to assess clinical utility. Patients who spent less than 24 h in the hospital were re‐included in the study for a sensitivity analysis in order to lessen the effect of survival bias. Variables having a missing rate of more than 20% would be eliminated, and the remaining missing values would be imputed using multiple imputation by chained equations (MICE) to avoid bias caused by missing data. All analyses used a significance threshold of *p* < 0.05.

## 3. Results

### 3.1. Comparison of Baseline Information

This study included 111 patients, with a mean age of 38.87 ± 1.06 years in the ARDS group and 37.18 ± 1.10 years in the non‐ARDS group. The cohort was predominantly male (*n* = 80, 72.07%) compared with female (*n* = 31, 27.93%). A total of 61 patients (54.95%) developed ARDS during the disease course. The 24 h IAP and length of ICU stay were significantly higher in patients in the ARDS cohort than in those in the non‐ARDS cohort (*p* < 0.05). In contrast, no statistically significant differences were observed in the remaining clinical parameters (*p* > 0.05) (Table 1).

**TABLE 1:** Comparison of baseline information.

Variables	ARDS (*N* = 61)	Non‐ARDS (*N* = 50)	*X* ^2^ */t/Z*	*p* value
Sex, *n* (%)			2.945	0.086
Male	48 (78.7)	32 (64.0)		
Female	13 (21.3)	18 (36.0)		
Age (mean ± SD)	38.87 ± 1.06	37.18 ± 1.10	−1.097	0.275
Hypertension, *n* (%)	18 (29.5)	12 (24.0)	0.423	0.516
Diabetes, *n* (%)	21 (34.4)	19 (38.0)	0.152	0.696
BMI (kg/m^2^, [mean ± SD])	28.68 ± 4.31	27.11 ± 3.95	−1.977	0.051
Number of pancreatitis episodes (M [*Q*_*L*_, *Q*_*U*_)]	1.00 (1.00, 2.00)	1.50 (1.00, 2.00)	−1.754	0.079
Smoking history, *n* (%)	12 (19.7)	11 (22.0)	0.091	0.763
Drinking history, *n* (%)	13 (21.3)	15 (30.0)	1.100	0.294
Combined DKA, *n* (%)	10 (16.4)	12 (24.0)	1.000	0.317
Blood purification, *n* (%)	23 (37.7)	19 (38.0)	0.001	0.975
Heart rate (mean ± SD)	115.66 ± 21.20	109.60 ± 20.51	−1.519	0.132
Random blood glucose (mmol/L, [mean ± SD])	12.86 ± 4.36	13.30 ± 4.94	0.499	0.619
24 h IAP [mmol/L, (M (*Q*_*L*_, *Q*_*U*_)]	15.00 (12.00, 19.00)	10.00 (8.00, 12.00)	−5.365	< 0.001
Length of ICU stay (M [*Q*_*L*_, *Q*_*U*_])	9.00 (6.00, 12.00)	4.00 (4.00, 6.00)	−5.583	< 0.001
24 h fluid balance (ml, (M [*Q*_*L*_, *Q*_*U*_])	1385.80 (107.65, 2757.00)	891.50 (288.65, 2194.95)	−0.771	0.441

*Note:* DKA, diabetic ketoacidosis.

Abbreviations: BMI, body mass index; IAP, intra‐abdominal pressure.

### 3.2. Comparison of Laboratory Test Indicators

Patients in the ARDS group exhibited comparatively lower levels of Ca^2+^ and greater levels of LDH, Lac, and IL‐6 than the non‐ARDS group (*p* < 0.05). The remaining 15 laboratory measures showed no discernible variations (*p* > 0.05) (Table 2).

**TABLE 2:** Comparison of laboratory test indicators.

Variables	ARDS (*N* = 61)	Non‐ARDS (*N* = 50)	*X* ^2^ */t/Z*	*p* value
WBC [× 10^9^/L, (M (*Q*_*L*_, *Q*_*U*_)]	12.30 (7.90, 16.15)	13.40 (10.78, 15.63)	−1.058	0.290
NEU [× 10^9^/L, (M (*Q*_*L*_, *Q*_*U*_)]	10.57 (17.98, 14.59)	11.92 (8.83, 16.70)	−1.476	0.140
24 h CRP [mg/L, (M (*Q*_*L*_, *Q*_*U*_)]	115.30 (40.30, 240.40)	109.75 (12.83, 230.90)	−1.144	0.253
48 h CRP [mg/L, (mean ± SD)]	192.20 ± 74.32	190.50 ± 84.5	−0.113	0.911
HCT [%, (mean ± SD)]	44.75 ± 7.10	44.63 ± 5.28	−0.101	0.920
PCT [ng/mL, (M (*Q*_*L*_, *Q*_*U*_)]	0.79 (0.26, 2.93)	0.48 (0.14, 2.17)	−1.713	0.087
IL‐6 [pg/mL, (M (*Q*_*L*_, *Q*_*U*_)]	151.6 (70.55, 339.6)	55.79 (11.97, 109.43)	−4.887	< 0.001
TNF‐*ɑ* [pg/mL, (M (*Q*_*L*_, *Q*_*U*_)]	2.80 (1.69, 4.45)	2.67 (1.92, 3.70)	−0.306	0.760
IFN‐*y* [pg/mL, (M (*Q*_*L*_, *Q*_*U*_)]	3.30 (2.50, 4.42)	2.68 (2.42, 4.23)	−1.395	0.163
Cr [μmol/L, (M (*Q*_*L*_, *Q*_*U*_)]	52.00 (40.50, 96.50)	52.50 (40.75, 66.00)	−0.883	0.377
TBIL [(μmol/L,M (*Q*_*L*_, *Q*_*U*_)]	9.70 (6.55, 15.30)	8.79 (6.78, 13.20)	−0.738	0.461
LDH [U/L, (M (*Q*_*L*_, *Q*_*U*_)]	397.00 (321.50, 564.00)	228.00 (160.25, 315.75)	−6.653	< 0.001
24 h TG [mmol/L, (mean ± SD)]	34.99 ± 24.73	37.21 ± 21.44	0.500	0.618
48 h TG [mmol/L, (M (*Q*_*L*_, *Q*_*U*_)]	9.16 (5.91, 14.48)	11.79 (6.41, 16.67)	0.374	0.374
Ca^2+^ [mmol/L, (M (*Q*_*L*_, *Q*_*U*_)]	1.97 (1.69, 2.10)	2.10 (1.93, 2.23)	2.155	0.005
PT [sec, (M (*Q*_*L*_, *Q*_*U*_)]	12.00 (11.35, 13.30)	11.70 (10.98, 12.73)	−1.619	0.105
APTT [sec, (M (*Q*_*L*_, *Q*_*U*_)]	28.10 (25.90, 32.40)	27.45 (25.18, 29.65)	−1.194	0.232
*D*‐dimer [μg/ml, (M (*Q*_*L*_, *Q*_*U*_)]	1.26 (0.40, 4.21)	0.77 (0.42, 1.86)	−1.603	0.109
Lac [mmol/L, (M (*Q*_*L*_, *Q*_*U*_)]	1.30 (1.00, 2.40)	1.00 (0.90, 1.40)	−3.086	0.002

*Note:* NEU, Neutrophil count; HCT, Hematocrit; PCT, Procalcitonin; IL‐6, Interleukin6; IFN, Interferon; Cr, Creatinine; TBIL, Total bilirubin; LDH, lactate dehydrogenase; TG, Triglyceride; Ca^2+^, Serum calcium; Lac, Lactic acid.

Abbreviations: APTT, Activated partial thromboplastin time; CRP, C‐reactive protein; PT, Prothrombin time; TNF, Tumor necrosis factor; WBC, White blood cell.

### 3.3. Comparison of Scoring Systems

Scoring comparisons of the two patient groups reveal that the ARDS group presented SOFA, APACHE II, and BISAP scores of 4.00 (3.00, 6.00), 15.52 ± 7.77, and 2.00 (1.00, 2.00), respectively. Moreover, the SOFA, APACHE II, and BISAP scores for the non‐ARDS group were 3.00 (2.00, 4.00), 11.90 ± 5.64, and 1.00 (1.00, 2.00), respectively. All three scores were significantly higher in the ARDS group compared to the non‐ARDS group (*p* < 0.05), as shown in Table 3.

**TABLE 3:** Comparison of scoring systems.

Variables	ARDS (*N* = 61)	Non‐ARDS (*N* = 50)	*X* ^2^ */t/Z*	*p* value
SOFA score [M (*Q*_*L*_, *Q*_*U*_)]	4.00 (3.00, 6.00)	3.00 (2.00, 4.00)	−4.347	< 0.001
APACHE II score (mean ± SD)	15.52 ± 7.77	11.90 ± 5.64	−2.843	0.005
BISAP score [M (*Q*_*L*_, *Q*_*U*_)]	2.00 (1.00, 2.00)	1.00 (1.00, 2.00)	−3.991	< 0.001

*Note:* APACHE II score, Acute Physiology and Chronic Health Evaluation score.

Abbreviations: BISAP score, Bedside Index of Severity in Acute Pancreatitis score; SOFA score, Sequential Organ Failure Assessment score.

### 3.4. Importance of Factors for ARDS Ranked by the Boruta Algorithm

**FIGURE 1:**
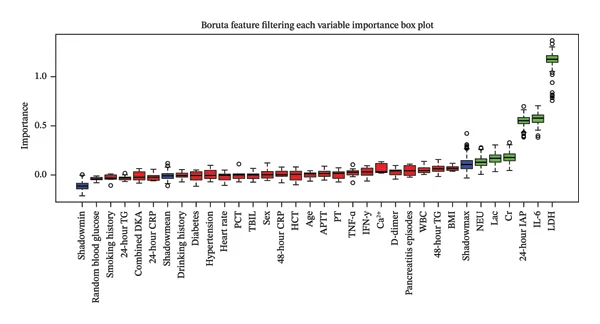
The Boruta test was used to rank the importance of potential risk factors for HTG‐AP‐associated ARDS. The horizontal axis lists the names of each variable, and the vertical axis represents the importance of each variable. Green boxes indicate important variables, red boxes indicate unimportant variables, and yellow boxes indicate potentially important variables.

Variables in the green region, such as NEU, Lac, Cr, 24 h IAP, IL‐6, and LDH, were shown to be significant elements in the Boruta algorithm report and are crucial to the model. Variables in the red region are non‐significant factors; variables in the yellow region are suspected factors that may be somewhat linked to a bad prognosis (not shown in this initial analysis). These Boruta‐validated factors are all obviously clinically relevant (Figure 1). All six of the variables that were chosen had VIFs of less than 5, which showed that multicollinearity was absent (Table S1). The filtered variables were incorporated into the multivariate logistic regression analysis. The findings indicated that 24 h IAP, IL‐6, and LDH were independent predictors of ARDS associated with HTG‐AP (*p* < 0.05) (Table 4).

**TABLE 4:** Multivariate logistic regression analysis results of independent predictors.

Predictive factor	Regression coefficient	SE	Wald	*p* value	OR	95% CI
24 h IAP	0.213	0.072	8.872	0.003	1.238	1.076 ∼ 1.424
IL‐6	0.005	0.002	5.402	0.020	1.005	1.001 ∼ 1.009
LDH	0.006	0.002	9.127	0.003	1.006	1.002 ∼ 1.011
NEU	0.001	0.012	0.011	0.915	1.001	0.978 ∼ 1.025
Lac	0.283	0.398	0.507	0.476	1.327	0.609 ∼ 2.893
Cr	−0.003	0.007	0.202	0.653	0.997	0.983 ∼ 1.011

*Note:* 24 hour IAP, IL‐6, and LDH were independent predictors of ARDS associated with HTG‐AP. Variables with *p* > 0.05 are shown for completeness but did not meet statistical significance as independent predictors.

### 3.5. Assessing the Projected Value

**FIGURE 2:**
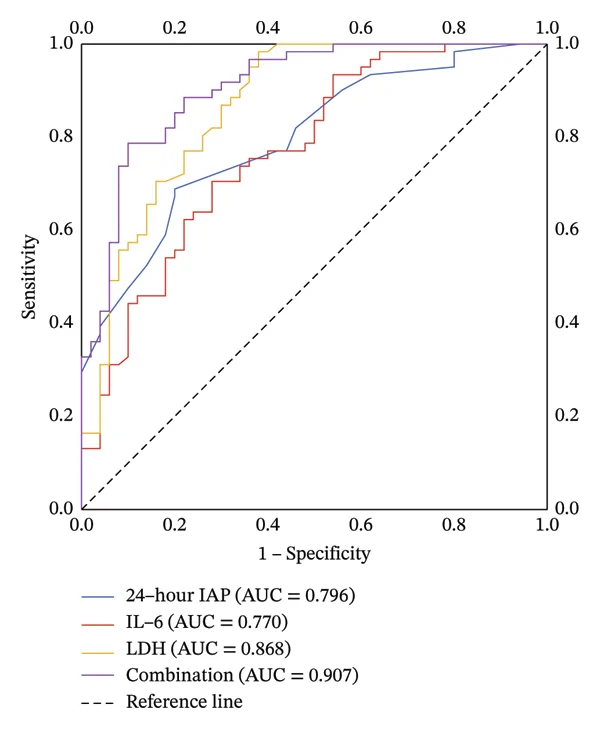
ROC curves of independent predictors and predictive models for HTG‐AP combined with ARDS. 24 h intra‐abdominal pressure (IAP), interleukin 6 (IL‐6), Lactate dehydrogenase (LDH), and area under the curve (AUC) of the predictive model ROC after combination were 0.796, 0.770, 0.868, and 0.907, respectively.

The ROC curve analysis revealed that, for 24 h IAP, AUC was 0.796, with a threshold of 12.25 mmHg, a sensitivity of 68.90%, and a specificity of 80.00%. For IL‐6, AUC was 0.770, with a threshold of 89.40 pg/mL, a sensitivity of 70.50%, and a specificity of 72.00%. For LDH, AUC was 0.868, with a threshold of 249.50 U/L, a sensitivity of 98.40%, and a specificity of 62.00% (all *p* < 0.05). When combined, the integrated model achieved an AUC of 0.907 (95% CI: 0.849–0.957), with a sensitivity of 78.70% and specificity of 90.00% (*p* < 0.05) (Table 5). These findings indicate strong predictive performance (Figure 2).

**TABLE 5:** ROC curves for each of the independent predictors and the combination.

Index	AUC	95% CI	*p*	Cut‐off value	Sensitivity (%)	Specificity (%)
24 h IAP	0.796	0.715 ∼ 0.877	< 0.001	12.25	68.90	80.00
IL‐6	0.770	0.683 ∼ 0.858	< 0.001	89.40	70.50	72.00
LDH	0.868	0.800 ∼ 0.936	< 0.001	249.50	98.40	62.00
Combination	0.907	0.849 ∼ 0.957	< 0.001		78.70	90.00

*Note:* IL‐6, interleukin6; LDH, lactate dehydrogenase; Combination, combine 24 h IAP; IL‐6; LDH. Cut‐off values were determined by maximizing the Youden index on ROC curves to optimize sensitivity and specificity.

Abbreviations: AUC, area under the curve; IAP, intra‐abdominal pressure.

### 3.6. Comparison of Predictive Models and Clinical Scoring Systems

**FIGURE 3:**
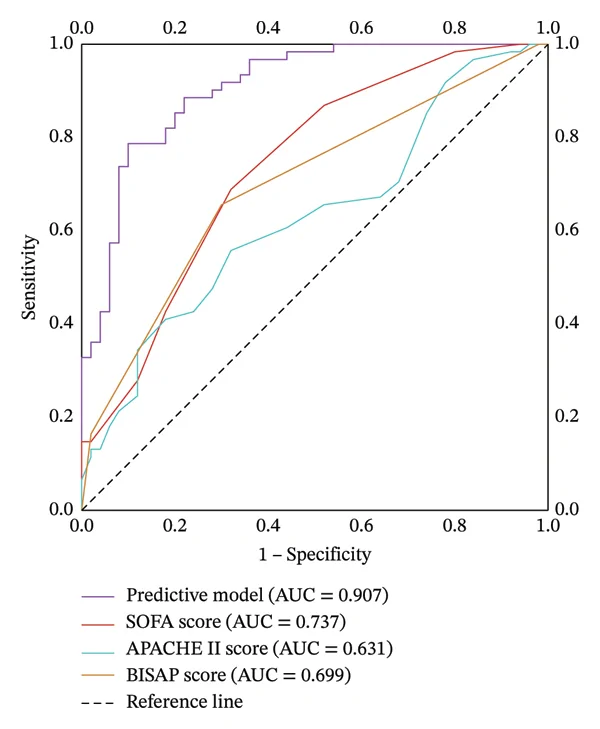
Comparison of ROC curves for the new predictive model and clinical scoring systems. The area under the curve (AUC) of the ROC curves for the predictive model, the Sequential Organ Failure Assessment (SOFA), the Acute Physiology and Chronic Health Evaluation (APACHE II), and the Bedside Index of Severity in Acute Pancreatitis (BISAP) scores were 0.907, 0.737, 0.631, and 0.699, respectively.

The prediction model has an AUC of 0.907, a sensitivity of 78.70%, and a specificity of 90.00%, according to ROC curve analysis. The SOFA score showed a sensitivity of 68.90%, a specificity of 68.00%, and an AUC of 0.737. The APACHE II score had an AUC of 0.631, a sensitivity of 55.70%, and a specificity of 68.00%. Furthermore, the BISAP score had an AUC of 0.699, a sensitivity of 65.60%, and a specificity of 98.00% (*p* < 0.05) (Table 6). The novel predictive model is equally therapeutically useful when compared to current clinical rating systems (Figure 3).

**TABLE 6:** ROC curves for predictive models and clinical scoring systems.

Index	AUC	95% CI	*p*	Sensitivity (%)	Specificity (%)
Predictive model	0.907	0.849 ∼ 0.957	< 0.001	78.70	90.00
SOFA score	0.737	0.644 ∼ 0.831	< 0.001	68.90	68.00
APACHE II score	0.631	0.528 ∼ 0.734	0.018	55.70	68.00
BISAP score	0.699	0.602 ∼ 0.797	< 0.001	65.60	98.00

*Note:* Predictive model, A predictive model comprising 24 h IAP; IL‐6, and LDH; SOFA score, Sequential Organ Failure Assessment score. APACHE II score, Acute Physiology and Chronic Health Evaluation score.

Abbreviation: AUC, area under the curve; BISAP score, Bedside Index of Severity in Acute Pancreatitis score.

### 3.7. Sensitivity Analysis for Patients With a Hospital Stay of Less Than 24 h

To address potential survivorship bias, we performed a sensitivity analysis that re‐included the three patients originally excluded due to hospital stay < 24 h (with multiple imputation for missing data). The analysis included 114 patients in total, 51 in the non‐ARDS group and 63 in the ARDS group. The results were consistent with the primary analysis: 24 h IAP, IL‐6, and LDH remained independent predictors, and the combined model achieved an AUC of 0.913 (95% CI: 0.861–0.965), comparable to the primary AUC of 0.907 (Table S1–S3) (Figure S1 and S2).

### 3.8. Regression Formula and Online Risk Calculator

The final prediction model for ARDS in HTG‐AP patients is as follows, based on the multivariate logistic regression analysis: Logit (*p*) = −5.448+0.213 × (IAP)+0.005 × (IL−6)+0.006 × (LDH), where LDH is expressed in U/L, IL‐6 in pg/mL, and IAP in mmHg. (*p* = 1)⁄((1 + e^{−Logit(p)})) is the anticipated likelihood of ARDS.

Based on the regression coefficients of three predictor variables, we created an online risk calculator. Please visit the following website for additional information: https://ardsprediction.streamlit.app/. In clinical practice, this calculator helps physicians perform individualized risk evaluations. A value near 1 means that there is a very high chance that this clinical outcome will occur under the present input conditions.

### 3.9. Internal Validation of the Model

**FIGURE 4:**
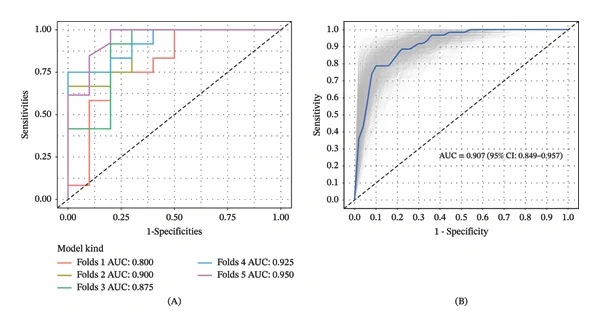
Internal validation of the predictive model using cross‐validation and bootstrap resampling. (A) The 5‐fold cross‐validation ROC curve illustrates the model’s ability to discriminate, and the mean AUROC is 0.890. (B) ROC curve of the integrated predictive model using 1,000 resamples for bootstrap internal validation. AUC appeared to be 0.907 (95% CI: 0.849–0.957). 0.894 was the bootstrap optimism‐corrected *C*‐index.

Five‐fold cross‐validation produced a mean accuracy of 0.784 and a mean AUROC of 0.890 (range: 0.800–0.950) (Figure 4A). Each validation fold included just about 22 patients due to the small overall sample size (*n* = 111). The variety in performance indicators (e.g., recall [sensitivity] ranging from 0.583 to 1.000) and the observed mean accuracy of 0.784 are typical of small‐sample cross‐validation and do not suggest poor model generalizability. Supporting Table S4 provides specific fold‐wise measurements. The bootstrap AUC was 0.907 (95% confidence interval: 0.849–0.957), and the C‐index was 0.894 after correction for optimism bias (Figure 4B). The outcomes confirm that the model is stable and does not exhibit substantial overfitting.

**FIGURE 5:**
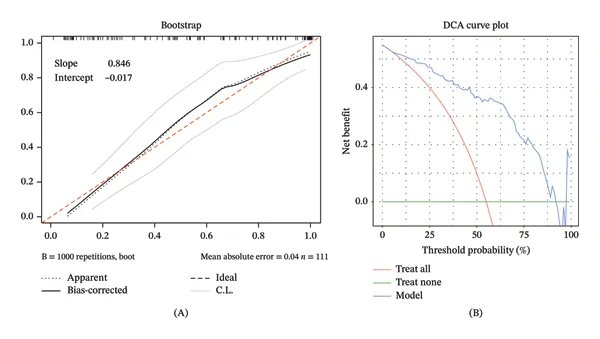
(A) Calibration curves for predictive models. (B) The DCA curve evaluation model’s clinical value.

The Hosmer‐Lemeshow test indicated that the model fit well (*p* = 0.16), and the calibration curve had a slope of 0.846 and an intercept of −0.017 (Figure 5A). The practical usefulness of this approach is further supported by DCA (Figure 5B), which shows positive net benefits over a range of threshold probabilities (Table S5).

## 4. Discussion

Over 50% of deaths in SAP are attributed to ARDS [12], with HTG‐AP patients demonstrating a more aggressive clinical course and higher mortality than biliary or alcoholic pancreatitis [3]. Our study cohort reflects this trend, with a mean age of 38.87 ± 1.06 years and a high ARDS incidence of 54.95%, underscoring HTG‐AP’s predilection for younger populations [13]. In addition, HTG‐AP patients in this cohort exhibited markedly elevated TG levels (24 h TG: 34.99 ± 2 4.73 mmol/L in the ARDS group, 37.21 ± 21.44 mmol/L in the non‐ARDS group) and interleukin‐6 levels (IL‐6: 151.6, 55.79 pg/mL), significantly higher than those reported in general ward populations with milder HTG‐AP (e.g., TG < 15 mmol/L, IL‐6 < 50 pg/mL) [14, 15]. This hyperinflammatory state, driven by free fatty acid–induced pancreatic autodigestion and systemic cytokine storms [16], predisposes patients to early organ dysfunction and necessitates intensive monitoring, e.g., IAP measurements and interventions such as blood purification. Existing predictive models for AP often lack ARDS‐specific risk stratification, highlighting the need for targeted research to improve early detection. By focusing on this high‐risk ICU subset of patients, this study aims to identify early predictors for ARDS in patients requiring advanced critical care. In this study, we identified 24 h IAP, IL‐6, and LDH as early independent predictors of ARDS in HTG‐AP patients.

24 h IAP: A Sentinel for Early Systemic Inflammation: Using the lateral bladder pressure method [17], we found that the 24‐h IAP (AUC = 0.796, threshold = 12.25 mmHg) was an early and robust predictor of ARDS, reflecting HTG‐AP’s rapid progression to IAH. Unlike prior studies focused on the 48 h IAP [18], our analysis emphasizes the clinical value of earlier measurement. IAH contributes to ARDS through mechanical compression (e.g., diaphragmatic elevation and reduced lung compliance) [19] and reflects systemic inflammatory burden [20]. Clinically, IAP‐guided strategies, including optimized fluid resuscitation and ventilator management, have reduced complication rates [21, 22], underscoring the importance of early monitoring within the first 24–48 h of admission. IAP of this study was assessed 24 h after admission, which may coincide with the duration of early preclinical ARDS in certain individuals, suggesting that reverse causation may be present. However, the time to ARDS onset in our cohort was 59.07 ± 31.83 h after admission to the EICU, indicating that the vast majority of cases had not yet developed ARDS at the time of IAP measurement. This is unlikely to have affected our results, even though ARDS itself may theoretically increase IAP through mechanisms like labored breathing and visceral congestion. Furthermore, we have investigated the pathophysiological connection between the development of ARDS and increased IAP. These results corroborate the direction of the connection found in this investigation.

IL‐6 and LDH: Core Biomarkers of Inflammation and Tissue Damage: IL‐6 (AUC = 0.770, threshold = 89.40 pg/mL) has emerged as a key inflammatory marker that links hypertriglyceridemia to cytokine storm formation via free fatty acid metabolism [23]. Our findings align with those of multicenter studies showing that an IL‐6 concentration ≥ 80 pg/mL predicts ARDS with ∼73% sensitivity [24]. IL‐6 activates *T*‐cell and hepatocyte inflammatory pathways, reinforcing its central role in AP progression [25]. LDH (AUC = 0.868, threshold = 249.50 U/L), a marker of cellular necrosis, exceeded thresholds established for ARDS in SAP patients [9] and is correlated with prolonged ICU stays and higher mortality [26, 27]. Elevated LDH in HTG‐AP likely reflects enhanced lipid peroxidation and multiorgan injury, which is consistent with its role in redox stress and inflammation [28, 29].

Clinical Utility of Predictive Models: Cutoff values for all predictors were determined by maximizing the Youden index and optimizing sensitivity and specificity. The combined model (AUC = 0.907) integrating IAP, IL‐6, and LDH achieved 78.70% sensitivity and 90% specificity (Table 5), outperforming individual markers. This supports its use in early triage and aligns with trends in precision medicine, where multimarker models enhance prognostic accuracy [30]. This model performs on par with or better than SOFA, APACHE II, and BISAP, and it has the added benefits of being user‐friendly and enabling earlier assessment. The results of a sensitivity analysis that included patients who were hospitalized for less than 24 h were consistent with the initial findings, suggesting that survival bias had no impact on our conclusions. The therapeutic usefulness of this model was further validated by internal validation and DCA. Although external validation is still necessary, these findings support the model’s dependability. Targeted intervention is still limited since few biomarkers or scoring methods allow for early ARDS detection in HTG‐AP. This research offers a workable solution. Our results indicate that the three indicators (24‐h IAP, IL‐6, and LDH) assessed within 24 h of admission can be used by physicians for early risk stratification of ARDS in HTG‐AP patients. Patients who have been classified as high‐risk may benefit from prompt supportive care and more regular monitoring, such as blood gas analysis, IAP monitoring, and respiratory assessments. While prior research has recommended therapies like IAP‐guided fluid resuscitation, early blood purification, and lung‐protective ventilation to enhance outcomes in comparable groups [7, 31], our model by itself does not directly require certain treatments. Before being put into practice, the model’s clinical value for directing certain therapy choices needs additional prospective validation and external verification. However, the model offers a useful tool for identifying high‐risk individuals who need to be monitored more closely and referred to critical care units as soon as possible.

Limitations and Future Directions: This study has several limitations. Regarding the generalizability of our results, our single‐center cohort consisted primarily of critically ill adult inpatients at a tertiary hospital in a specific region; one inherent limitation of single‐center studies is that patients from primary care facilities and medical institutions in other regions may be underrepresented in the sample. Additionally, due to the inclusion of only EICU patients, the risk of ARDS in non‐ICU settings may be underestimated. Moreover, the model’s performance may be underestimated in populations with earlier access to critical care, given the median 24 h delay from symptom onset to EICU admission. Methodological constraints include a relatively small sample size and delayed cytokine profiling. Future studies should focus on larger, multicenter, prospective cohorts to enhance generalizability. Integrating advanced approaches such as spatial transcriptomics [32] and AI‐driven analytics [30, 33] may refine early ARDS risk prediction. Additionally, therapeutic therapies for lipid metabolism, e.g., PCSK9 inhibitors [34, 35] and redox signaling [36], hold promise for preventing ARDS in HTG‐AP patients.

## 5. Conclusions

This study establishes a robust predictive framework for ARDS in HTG‐AP patients, incorporating mechanical (IAP), inflammatory (IL‐6), and necrosis‐related (LDH) indicators. By addressing both statistical rigor and clinical relevance, these findings support early risk stratification and targeted interventions. Further confirmation through large‐scale, multicenter, prospective studies and in‐depth biological research is essential to turn these findings into practical tools that enhance care and outcomes for HTG‐AP patients at risk of ARDS.

## Author Contributions

Kaiqiang Zhu: data gathering, resource collection, and major writing draft.

Bin Lu: data gathering, resource collection, and assisted writing draft.

Ranran Zhou: analyzed the data and assisted writing draft.

Lei Xu: research design, revision, and correspondence.

## Funding

This research received no external funding.

## Disclosure

The final manuscript was read and approved by all writers.

## Ethics Statement

The Ethics Committee of Affiliated Hospital of XuZhou University authorized the study protocol (No.XYFY2025‐KL349‐01). The requirement for informed consent was waived by the Ethics Committee of Affiliated Hospital of XuZhou Medical University.

## Conflicts of Interest

The authors declare no conflicts of interest.

## Supporting Information

Additional supporting information can be found online in the Supporting Information section.

## Supporting information


**Supporting Information 1** Completed TRIPOD checklist of the prediction model in accordance with TRIPOD recommendations.

**Supporting Information 2** Completed STROBE checklist of the observational study in accordance with STROBE guidelines.

**Supporting Information 3** Includes: Table S1 (multicollinearity tests using variance inflation factor), Table S2 (multivariate logistic regression results for the sensitivity analysis re‐including patients with < 24 h hospital stay, with multiple imputation), Table S3 (ROC curve analysis for the sensitivity analysis), Table S4 (performance metrics for 5‐fold cross‐validation of the predictive model), Table S5 (probabilistic net benefit of the predictive model at different threshold probabilities), Figure S1 (Boruta algorithm variable importance ranking after re‐including patients with < 24 h hospital stay), and Figure S2 (ROC curves for the predictive models in the sensitivity analysis).

## Data Availability

The authors state that all of the data used to support the study’s findings are included in the manuscript and that any raw data can be requested from the corresponding author.
