# Supplementary material for: Lactate Dehydrogenase, Interleukin‐6, and 24 h Intra‐Abdominal Pressure Exhibit Early Predictive Value in Hypertriglyceridemic Pancreatitis Complicated by Acute Respiratory Distress Syndrome
Source: Emerg Med Int. 2026 Sep 2;2026:8368299. doi: 10.1155/emmi/8368299 (PMC13535855; doi:10.1155/emmi/8368299)
Supplement: Supplementary file 3 [file EMMI-2026-8368299-s003.docx]

**Table S1: Multicollinearity tests**

| Variables | VIF | 1/VIF |
| --- | --- | --- |
| LDH | 2.202 | 0.495 |
| IL-6 | 1.652 | 0.605 |
| 24-hour IAP | 1.186 | 0.843 |
| NEU | 1.072 | 0.933 |
| Lac | 1.732 | 0.577 |
| Cr | 2.153 | 0.464 |
| Mean VIF | 1.666 | |

**Table S2. Multivariate logistic regression for sensitivity analysis**

| Predicyive factor | Regression  coefficient | SE | Wald | *P-*value | OR | 95%CI |
| --- | --- | --- | --- | --- | --- | --- |
| 24-hour IAP | 0.179 | 0.072 | 6.161 | 0.013 | 1.196 | 1.038～1.379 |
| IL-6 | 0.006 | 0.002 | 6.431 | 0.011 | 1.006 | 1.001～1.010 |
| LDH | 0.007 | 0.002 | 9.600 | 0.002 | 1.007 | 1.002～1.011 |
| BMI | 0.107 | 0.065 | 2.768 | 0.096 | 1.113 | 0.981 ~ 1.264 |
| Lac | 0.138 | 0.417 | 0.110 | 0.740 | 1.148 | 0.507 ~ 2.602 |
| Cr | -0.005 | 0.007 | 0.465 | 0.495 | 0.995 | 0.981～1.009 |

**Table S3. ROC Curve for Sensitivity Analysis**

| Index | AUC | 95%CI | *P* | Cut-off value | Sensitivity(%) | Specificity(%) |
| --- | --- | --- | --- | --- | --- | --- |
| 24-hour IAP | 0.791 | 0.710～0.872 | <0.001 | 12.25 | 68.30 | 80.40 |
| IL-6 | 0.763 | 0.676～0.850 | <0.001 | 89.40 | 69.80 | 70.60 |
| LDH | 0.875 | 0.809～0.940 | <0.001 | 249.5 | 98.40 | 62.70 |
| Combination | 0.913 | 0.861～0.965 | <0.001 |  | 77.80 | 92.20 |

*AUC: Area under the curve; IAP: Intra-abdominal pressure ;IL-6: Interleukin6; LDH: Lactate dehydrogenase; Combination: Combine 24-hour IAP, IL-6, LDH. Cut-off values were determined by maximizing the Youden index on ROC curves to optimize sensitivity and specificity.*

**Table S4. Performance Metrics for 5-Fold Cross-Validation of the Predictive Model**

| **Fold** | **Accuracy** | **AUROC** | **Recall** | **Specificity** | **F1-Score** | **MCC** |
| --- | --- | --- | --- | --- | --- | --- |
| Fold 1 | 0.682 | 0.800 | 0.583 | 0.800 | 0.667 | 0388 |
| Fold 2 | 0.818 | 0.900 | 0.917 | 0.700 | 0.846 | 0.638 |
| Fold 3 | 0.864 | 0.875 | 1.000 | 0.700 | 0.889 | 0.748 |
| Fold 4 | 0.818 | 0.925 | 0.917 | 0.700 | 0.846 | 0.638 |
| Fold 5 | 0.739 | 0.950 | 0.615 | 0.900 | 0.727 | 0.524 |
| Mean | 0.784 | 0.890 | 0.806 | 0.760 | 0.795 | 0.587 |

*AUROC = area under the receiver operating characteristic curve; MCC = Matthews correlation coefficient; F1-Score = harmonic mean of precision and recall. Performance metrics are reported for each fold and the overall mean to demonstrate internal model stability.*

**Table S5. Probabilistic net profit of the predictive model at different thresholds**

| Threshold | Predictor | All | None |
| --- | --- | --- | --- |
| 0.01 | 0.544999544999545 | 0.544999544999545 | 0 |
| 0.02 | 0.540540540540541 | 0.540356683213826 | 0 |
| 0.03 | 0.535896721463732 | 0.535618092319123 | 0 |
| 0.04 | 0.531156156156156 | 0.530780780780781 | 0 |
| 0.05 | 0.526789947842579 | 0.525841631104789 | 0 |
| 0.06 | 0.522522522522523 | 0.520797393137819 | 0 |
| 0.07 | 0.521069456553328 | 0.515644676935 | 0 |
| 0.08 | 0.517430473952213 | 0.510379945162554 | 0 |
| 0.09 | 0.516582516582517 | 0.504999504999505 | 0 |
| 0.1 | 0.514514514514515 | 0.4994994994995 | 0 |
| 0.11 | 0.511691466747647 | 0.49387589837028 | 0 |
| 0.12 | 0.509009009009009 | 0.488124488124488 | 0 |
| 0.13 | 0.50647198923061 | 0.482240861551206 | 0 |
| 0.14 | 0.502618897967735 | 0.476220406452965 | 0 |
| 0.15 | 0.500264970853206 | 0.470058293587705 | 0 |
| 0.16 | 0.4997854997855 | 0.463749463749464 | 0 |
| 0.17 | 0.497883425594269 | 0.45728861391512 | 0 |
| 0.18 | 0.487145682267633 | 0.4506701823775 | 0 |
| 0.19 | 0.48559670781893 | 0.443888332777222 | 0 |
| 0.2 | 0.486486486486487 | 0.436936936936937 | 0 |
| 0.21 | 0.483065343824838 | 0.429809556391835 | 0 |
| 0.22 | 0.484638484638485 | 0.422499422499423 | 0 |
| 0.23 | 0.481338481338481 | 0.414999414999415 | 0 |
| 0.24 | 0.471787577050735 | 0.407302038880986 | 0 |
| 0.25 | 0.471471471471472 | 0.3993993993994 | 0 |
| 0.26 | 0.468224981738495 | 0.391283175066959 | 0 |
| 0.27 | 0.45353572750833 | 0.382944588424041 | 0 |
| 0.28 | 0.45045045045045 | 0.374374374374374 | 0 |
| 0.29 | 0.447278264179673 | 0.365562745844436 | 0 |
| 0.3 | 0.444015444015444 | 0.356499356499357 | 0 |
| 0.31 | 0.440658049353702 | 0.347173260216739 | 0 |
| 0.32 | 0.441441441441441 | 0.337572866984632 | 0 |
| 0.33 | 0.429070861906683 | 0.327685894850074 | 0 |
| 0.34 | 0.425607425607426 | 0.317499317499318 | 0 |
| 0.35 | 0.422037422037422 | 0.306999306999307 | 0 |
| 0.36 | 0.423423423423423 | 0.296171171171171 | 0 |
| 0.37 | 0.425139425139425 | 0.284999284999285 | 0 |
| 0.38 | 0.41267073525138 | 0.273467015402499 | 0 |
| 0.39 | 0.409097622212376 | 0.261556638605819 | 0 |
| 0.4 | 0.411411411411411 | 0.249249249249249 | 0 |
| 0.41 | 0.398839517483585 | 0.236524660253474 | 0 |
| 0.42 | 0.395153774464119 | 0.22336129232681 | 0 |
| 0.43 | 0.391338707128181 | 0.209736051841315 | 0 |
| 0.44 | 0.394465894465895 | 0.195624195624196 | 0 |
| 0.45 | 0.390663390663391 | 0.180999180999181 | 0 |
| 0.46 | 0.402068735402069 | 0.165832499165833 | 0 |
| 0.47 | 0.380588135305116 | 0.150093489716131 | 0 |
| 0.48 | 0.376299376299376 | 0.133749133749134 | 0 |
| 0.49 | 0.363893305069776 | 0.116763822646176 | 0 |
| 0.5 | 0.369369369369369 | 0.0990990990990992 | 0 |
| 0.51 | 0.366059937488509 | 0.0807133664276522 | 0 |
| 0.52 | 0.362612612612613 | 0.0615615615615616 | 0 |
| 0.53 | 0.350009584052137 | 0.0415947862756374 | 0 |
| 0.54 | 0.358401880141011 | 0.0207598903251077 | 0 |
| 0.55 | 0.355355355355355 | -0.001001001 | 0 |
| 0.56 | 0.352170352170352 | -0.023751024 | 0 |
| 0.57 | 0.363712549759061 | -0.047559187 | 0 |
| 0.58 | 0.361218361218361 | -0.072501073 | 0 |
| 0.59 | 0.353548670621841 | -0.098659635 | 0 |
| 0.6 | 0.351351351351351 | -0.126126126 | 0 |
| 0.61 | 0.349041349041349 | -0.155001155 | 0 |
| 0.62 | 0.346609767662399 | -0.185395922 | 0 |
| 0.63 | 0.344046749452155 | -0.21743365 | 0 |
| 0.64 | 0.341341341341341 | -0.251251251 | 0 |
| 0.65 | 0.329472329472329 | -0.287001287 | 0 |
| 0.66 | 0.317435082140964 | -0.324854266 | 0 |
| 0.67 | 0.305214305214305 | -0.365001365 | 0 |
| 0.68 | 0.292792792792793 | -0.407657658 | 0 |
| 0.69 | 0.289160127869805 | -0.453065969 | 0 |
| 0.7 | 0.276276276276276 | -0.501501502 | 0 |
| 0.71 | 0.245107176141659 | -0.553277415 | 0 |
| 0.72 | 0.240669240669241 | -0.608751609 | 0 |
| 0.73 | 0.226893560226894 | -0.668335002 | 0 |
| 0.74 | 0.221760221760222 | -0.732501733 | 0 |
| 0.75 | 0.216216216216216 | -0.801801802 | 0 |
| 0.76 | 0.201201201201201 | -0.876876877 | 0 |
| 0.77 | 0.224833529181355 | -0.958480219 | 0 |
| 0.78 | 0.21048321048321 | -1.047502048 | 0 |
| 0.79 | 0.204633204633205 | -1.145002145 | 0 |
| 0.8 | 0.189189189189189 | -1.252252252 | 0 |
| 0.81 | 0.182076813655761 | -1.370791844 | 0 |
| 0.82 | 0.174174174174174 | -1.502502503 | 0 |
| 0.83 | 0.165341812400636 | -1.649708532 | 0 |
| 0.84 | 0.146396396396396 | -1.815315315 | 0 |
| 0.85 | 0.117117117117117 | -2.003003003 | 0 |
| 0.86 | 0.0952380952380952 | -2.217503218 | 0 |
| 0.87 | 0.0713790713790714 | -2.465003465 | 0 |
| 0.88 | 0.045045045045045 | -2.753753754 | 0 |
| 0.89 | 0.0155610155610155 | -3.095004095 | 0 |
| 0.9 | 0.054054054054054 | -3.504504505 | 0 |

**FIGURE S1.** After including patients with a hospital stay of less than 24 hours, the Boruta algorithm was used to rank the variables based on the importance of each feature.

**FIGURE** **S2.** ROC curves for the predictive models in the sensitivity analysis. The area under the curve (AUC) for the 24-hour intra-abdominal pressure (IAP), interleukin-6 (IL-6), lactate dehydrogenase (LDH), and combined predictive models was 0.791, 0.875, 0.763, and 0.907, respectively.
